# Serotonin 5-HT_2C_ Receptor Cys23Ser Single Nucleotide Polymorphism Associates with Receptor Function and Localization *In Vitro*

**DOI:** 10.1038/s41598-019-53124-2

**Published:** 2019-11-13

**Authors:** Michelle A. Land, Holly L. Chapman, Brionna D. Davis-Reyes, Daniel E. Felsing, John A. Allen, F. Gerard Moeller, Lisa A. Elferink, Kathryn A. Cunningham, Noelle C. Anastasio

**Affiliations:** 1 Center for Addiction Research, Galveston, TX USA; 2 Department of Pharmacology and Toxicology, Galveston, TX USA; 3 Department of Neuroscience, Cell Biology and Anatomy, University of Texas Medical Branch, Galveston, TX USA; 4 Department of Psychiatry, Virginia Commonwealth University, Richmond, VA USA

**Keywords:** Endosomes, Glycosylation, Cellular neuroscience, Genetics of the nervous system

## Abstract

A non-synonymous single nucleotide polymorphism of the human serotonin 5-HT_2C_ receptor (5-HT_2C_R) gene that converts a cysteine to a serine at amino acid codon 23 (Cys23Ser) appears to impact 5-HT_2C_R pharmacology at a cellular and systems level. We hypothesized that the Cys23Ser alters 5-HT_2C_R intracellular signaling via changes in subcellular localization *in vitro*. Using cell lines stably expressing the wild-type Cys23 or the Ser23 variant, we show that 5-HT evokes intracellular calcium release with decreased potency and peak response in the Ser23 versus the Cys23 cell lines. Biochemical analyses demonstrated lower Ser23 5-HT_2C_R plasma membrane localization versus the Cys23 5-HT_2C_R. Subcellular localization studies demonstrated O-linked glycosylation of the Ser23 variant, but not the wild-type Cys23, may be a post-translational mechanism which alters its localization within the Golgi apparatus. Further, both the Cys23 and Ser23 5-HT_2C_R are present in the recycling pathway with the Ser23 variant having decreased colocalization with the early endosome versus the Cys23 allele. Agonism of the 5-HT_2C_R causes the Ser23 variant to exit the recycling pathway with no effect on the Cys23 allele. Taken together, the Ser23 variant exhibits a distinct pharmacological and subcellular localization profile versus the wild-type Cys23 allele, which could impact aspects of receptor pharmacology in individuals expressing the Cys23Ser SNP.

## Introduction

Serotonin (5-HT) is an important neurotransmitter involved in a myriad of physiological and psychological functions. The actions of 5-HT are mediated by a family of 14 5-HT receptors (5-HT_X_Rs) based upon structural and functional characteristics (5-HT_1_R – 5-HT_7_R) (for review^1^). The 5-HT_2C_R receptor is a G protein-coupled receptor (GPCR) and the canonical G protein-dependent signaling through the 5-HT_2C_R is engendered by 5-HT-stimulated coupling predominantly to Gα_q/11_ to activate the enzyme phospholipase C_β_ (PLC_β_) which generates phosphoinositide hydrolysis (IP_3_) and intracellular calcium (${{{\rm{Ca}}}_{i}}^{++}$) mobilization^2^. The 5-HT_2C_R also signals through other second messengers which can include phospholipase D (PLD) and phospholipase A_2_ (PLA_2_), cyclic nucleotides, and extracellular signal-regulated kinases (ERK_1/2_)^3^, providing a diverse landscape of available signaling cascades and mechanisms. Serotonin neurotransmission through the 5-HT_2C_R within corticolimbic circuitry is implicated in Parkinson’s disease^4^, depression^5^, suicide^6,7^, schizophrenia^8^, obesity^9,10^, and is a critical driver of cognitive and/or behavioral dimensions underlying relapse-related behaviors in cocaine use disorder^11–14^. Thus, there is a critical role for the 5-HT_2C_R system in neuropsychiatric disorders and a greater neurobiological understanding of the interface between the 5-HT_2C_R system and disease processes may lead to the design of new targeted diagnostic and pharmacotherapeutic strategies.

Single nucleotide polymorphisms (SNPs) of the human 5-HT_2C_R gene (*HTR2C*), located on the X chromosome, associate with behavioral phenotypes, psychiatric conditions, and response to psychiatric medications including atypical antipsychotics and antidepressants^15–22^. There are four widely studied *HTR2C* SNPs, three within the promotor and 5′ untranslated region, which could affect gene expression^20^. The fourth SNP is a non-synonymous SNP that converts a cysteine (Cys) to a serine (Ser) at amino acid codon 23 in the N-terminal extracellular domain (Cys23Ser; rs6318)^23^. The prevalence of the Cys23Ser SNP ranges from 30% in populations with African ancestral informative markers to 12% in populations with European ancestral informative markers (1000Genome). The Ser23 variant associates with major depression, bipolar disorder, borderline personality disorder^17–19^, and high sensitivity to drug-associated cues (cue reactivity) in cocaine users^24^ versus the wild-type Cys23. Further, the Ser23 associates with an altered response to antidepressants and atypical antipsychotics^15,25,26^. Although the aforementioned association studies have investigated the Ser23 in neuropsychiatric disorders, much remains to be learned concerning the impact of this SNP on cellular function^21,23,27–29^.

The Cys23Ser SNP may impact phenotypic behaviors and cellular function through alterations in the structural integrity of the 5-HT_2C_R protein, the efficiency of 5-HT_2C_R ligands and signal transduction mechanisms and/or receptor subcellular localization profiles^30^. The few studies that have investigated the functional significance of the Cys23Ser SNP *in vitro* demonstrate altered sensitivity to 5-HT_2C_R ligands and changes in intracellular signaling properties^27,29^. *In vivo* rodent studies indicate lower 5-HT_2C_R function and shift in the subcellular localization profile of the 5-HT_2C_R in high cue reactivity to cocaine^13,14^. Localization of the 5-HT_2C_R at the plasma membrane is a tightly regulated process and essential for receptor function^31,32^. GPCRs are synthesized, folded and glycosylated in the endoplasmic reticulum and Golgi apparatus, and following proper maturation trafficked through the secretory pathway to the plasma membrane^33,34^. Upon stimulation, the 5-HT_2C_R undergoes agonist-induced desensitization by phosphorylation of its C-terminus^31^ by G protein receptor kinase 2 resulting in a disassociation from the G-protein and association with β-arrestin^35^. Following agonist-mediated receptor endocytosis, the 5-HT_2C_R can be resensitized and sent back to the plasma membrane from the early endosomes or recycling endosomes^32,35–37^. These pathways are integral steps in GPCR function, however the actual impact of the Cys23Ser SNP on 5-HT_2C_R subcellular localization, particularly at the plasma membrane, is unknown.

Here, we tested the hypothesis that the Cys23Ser SNP fundamentally alters 5-HT_2C_R functional capacity via changes in receptor subcellular localization profiles. We interrogated the pharmacogenetic impact of the Cys23Ser SNP on 5-HT_2C_R functional capacity using a series of *in vitro* biotechniques (${{{\rm{Ca}}}_{i}}^{++}$ release, immunocytochemistry, Wes^TM^ automated immunoblotting, radioligand binding, surface biotinylation) to demonstrate that the Ser23 variant attenuates agonist-induced intracellular signaling and basally has lower plasma membrane expression with a distinct localization pattern within the recycling pathway than the wild-type Cys23.

## Results

### The Cys23Ser SNP alters the functional response of the 5-HT_2C_R to 5-HT

Most signaling studies focused on GPCRs utilize immortal mammalian cell lines as these are easily manipulated, allow for better control of expression levels of the gene of interest, and are straightforwardly amenable to bioresponsive and subcellular localization assays. We employed RNAseq analyses to demonstrate that CHO cell lines express some of the major players in 5-HT_2C_R localization and signaling, including Camk1 (Calmodulin)^38,39^, Pten (PTEN, phosphatase and tensin homolog)^40,41^, and low levels of Dlg4 (PSD95, postsynaptic density 95)^32^ (unpublished observations). We engineered CHOp38 cells^42^ (CHO cells expressing synaptophysin/p38, see Methods for details on the generation of the cell line) to stably express the human Cys23 allele or the Ser23 allele of the non-edited (INI) 5-HT_2C_R. During the generation of our stable cell lines we were able to select for 35 Cys23-expressing clones and one Ser23-expressing clone. Each clone was evaluated for total 5-HT_2C_R protein expression using the Wes^TM^ automated Western blotting system. Three Cys23 5-HT_2C_R CHOp38 clones were selected: one with equal 5-HT_2C_R expression (Cys23 Clone 1) to the Ser23 5-HT_2C_R CHOp38 cell line, one with 5-HT_2C_R expression greater than Cys23 Clone 1 (Cys23 Clone 2) and one with 5-HT_2C_R expression lower than Cys23 Clone 1 (Cys23 Clone 3). As shown in Supplementary Fig. 1, there was a concentration-dependent increase in ${{{\rm{Ca}}}_{i}}^{++}$ levels following 5-HT administration in all four clones. The 5-HT peak response for the Ser23 (E_max_ = 57.62 ± 14.83%) was ~43% lower relative to Cys23 Clone 1 (E_max_ = 101.4 ± 19.16%). The Cys23 Clone 2 had a 36.4% higher 5-HT peak response (E_max_ = 137.8 ± 19.95%) while Cys23 Clone 3 demonstrated a 9.46% decrease in 5-HT peak response (E_max_ = 91.94 ± 4.56%) versus Cys23 Clone 1. The chosen Cys23 (Clone 1) and Ser23 lines with equal levels of total 5-HT_2C_R protein were employed for all additional analyses presented herein.

**Figure 1:**
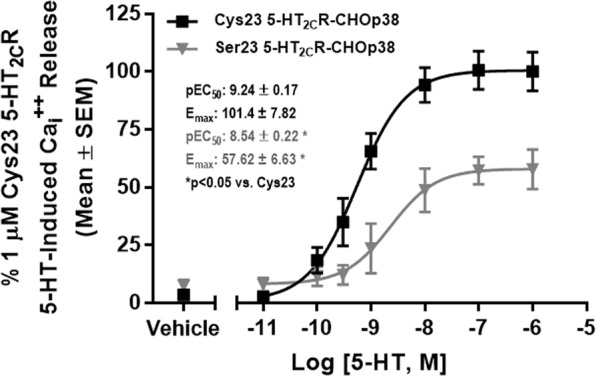
Serotonin-induced intracellular calcium release is lower in the Ser23-CHOp38 vs. Cys23-CHOp38 stably expressing cell lines. Average traces of a 5-HT induced concentration-dependent ${{{\rm{Ca}}}_{i}}^{++}$ release in Cys23 and Ser23 5-HT_2C_R-CHOp38 cells (normalized to the average 1 µM 5-HT response in the Cys23 5-HT_2C_R-CHOp38 to give a percent response). The pEC_50_ and E_max_ (mean ± SEM) were from 5 to 6 individual experiments. *p < 0.05 vs. Cys23 5-HT_2C_R-CHOp38.

To test the hypothesis that the Cys23Ser SNP alters 5-HT_2C_R-mediated signaling release relative to the wild-type 5-HT_2C_R, we assessed ${{{\rm{Ca}}}_{i}}^{++}$ release^40^. As shown in Fig. 1, there was a concentration-dependent increase in ${{{\rm{Ca}}}_{i}}^{++}$ levels following 5-HT administration in both the Cys23 5-HT_2C_R-CHOp38 and Ser23 5-HT_2C_R-CHOp38 cells. The Ser23 allele-expressing cells (Fig. 1, pEC_50_ = 8.54 ± 0.22) displayed a lower potency versus the Cys23 allele-expressing cells (Fig. 1, pEC_50_ = 9.24 ± 0.17; t(9) = 2.56, p < 0.05). These data are consistent with previous reports showing a shift in the potency of the Ser23 allele relative to the Cys23 allele^27,29,43^. The 5-HT peak response in the Ser23 allele-expressing cells (Fig. 1; E_max_ = 57.62 ± 6.63%) was ~43% lower relative to the Cys23 allele-expressing cells (Fig. 1; E_max_ = 101.4 ± 7.82%; t(9) = 4.16, p < 0.05). As expression of the Ser23 allele results in the loss of the single cysteine residue in the N-terminus, the removal of the Cys-dependent disulfide bond formation may alter the conformation of the 5-HT_2C_R and ultimately impact the potency of 5-HT to activate the Ser23 variant^43,44^.

**Figure 2:**
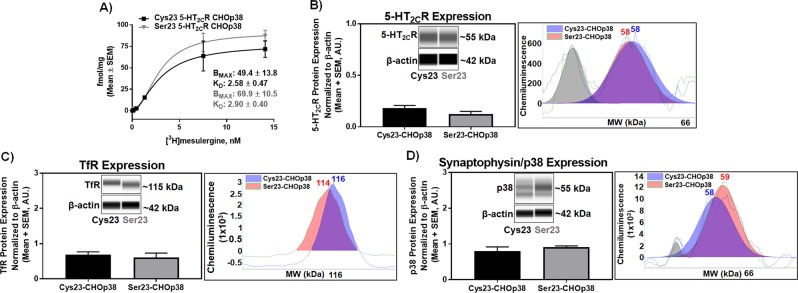
Total 5-HT_2C_R protein levels do not differ between the Cys23- and Ser23-CHOp38 cell lines. **(A)** Representative saturation binding isotherm of stably transfected Cys23 and Ser23 5-HT_2C_R-CHOp38 cells. Results obtained for K_D_ and B_max_ values were from 3 independent experiments. Total homogenate **(B)** 5-HT_2C_R (n.s.; n = 7), **(C)** TfR (n.s.; n = 3) and **(D)** p38 (n.s.; n = 6) protein expression normalized to β-actin in stably expressing Cys23 and Ser23 5-HT_2C_R-CHOp38 cells. Representative virtual blot-like images (left) and electropherograms adjusted to baseline (right) were generated by the Simple Western Compass software using the area under the curve for the peak of interest. Full length electropherograms and virtual blot-like images are located in Supplementary Figure 2.

This pharmacological profile was observed despite the fact that total 5-HT_2C_R protein expression between the Cys23 allele- and Ser23 allele-expressing cells was comparable (Fig. 2A). Saturation radioligand binding assays revealed that the Cys23 and Ser23 5-HT_2C_R-CHO-p38 cell lines stably express similar levels of 5-HT_2C_R (B_MAX_ = 49.4 ± 13.8 and 69.9 ± 10.5 fmol/mg protein, for the Cys23 and Ser23 5-HT_2C_R-CHO-p38 respectively; n.s.) (Fig. 2A). Additionally, both cell lines exhibited similar affinity (K_D_) for [^3^H]-mesulergine of 2.58 ± 0.47 and 2.90 ± 0.40 nM for the Cys23 and Ser23 5-HT_2C_R-CHO-p38 cell lines, respectively (n.s.) (Fig. 2A). Total protein expression of the 5-HT_2C_R as assessed using the Wes^TM^ automated Western blotting system showed no significant difference between the Cys23 and Ser23 5-HT_2C_R-CHOp38 cells (Fig. 2B and Supplementary Fig. 2A; n.s.). Stable transfection of the Cys23 allele or Ser23 allele into the CHOp38 cells did not alter total transferrin receptor (TfR) (Fig. 2C and Supplementary Fig. 2C, n.s.) or synaptophysin/p38 (Fig. 2D and Supplementary Fig. 2D, n.s.) protein expression levels. We propose that the lower maximal 5-HT-induced ${{{\rm{Ca}}}_{i}}^{++}$ response by the Ser23 variant may be due to differences in plasma membrane localization of the 5-HT_2C_R as the subcellular localization can fundamentally control the signaling efficacy and specificity of GPCRs.

### Cys23Ser SNP lowers 5-HT_2C_R plasma membrane localization

**Figure 3:**
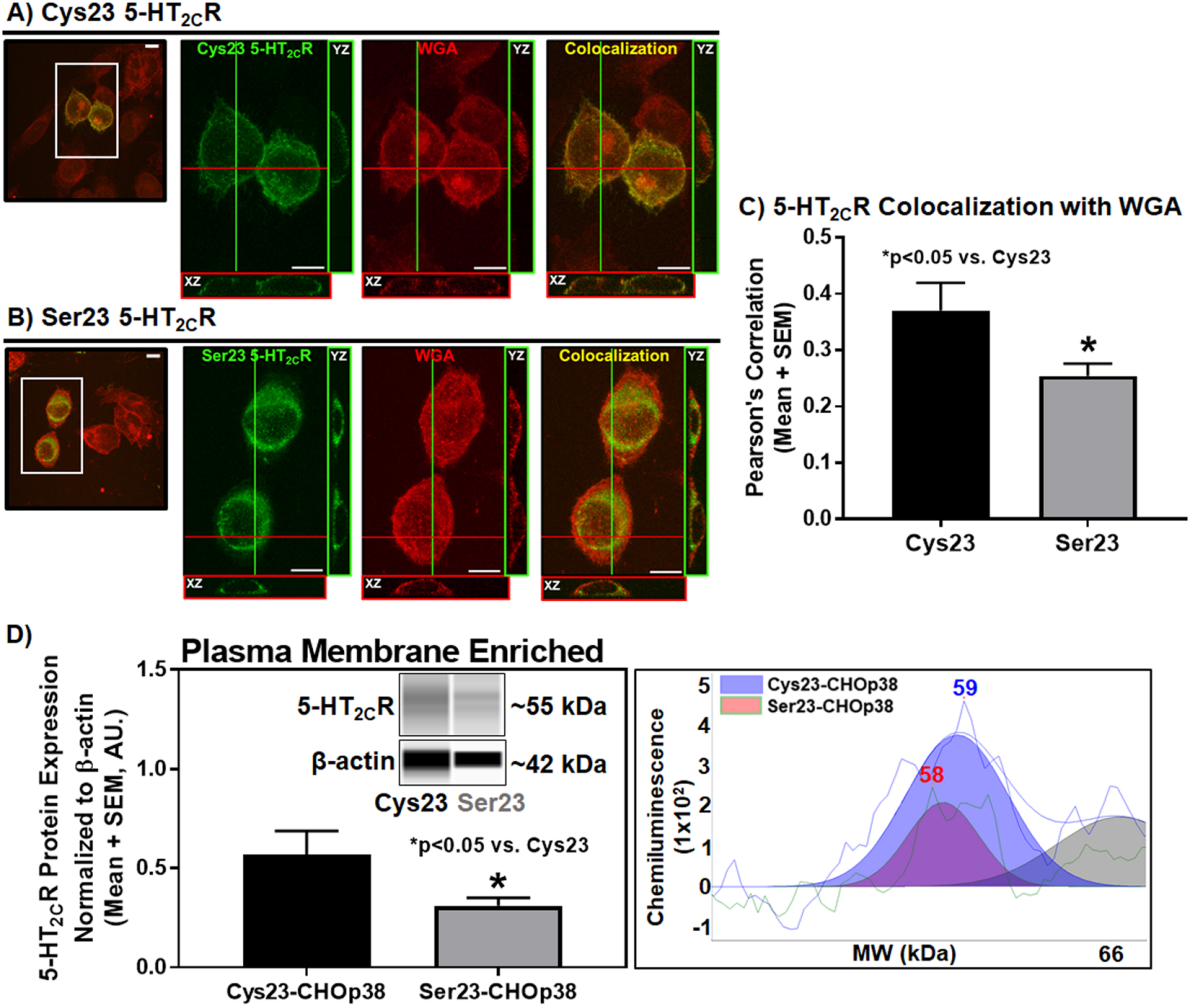
Plasma membrane 5-HT_2C_R protein is lower in Ser23- vs. Cys23-CHOp38 cell lines. Colocalization of transiently transfected CHOp38 cells expressing **(A)** Cys23 or **(B)** Ser23 5-HT_2C_R in green with the plasma membrane marker wheat germ agglutinin (WGA) in red. Colocalization images were prepared from a confocal series of Cys23 or Ser23 5-HT_2C_R transiently transfected CHOp38 cells sectioned tangentially. Orthogonal views demonstrate Cys23 or Ser23 5-HT_2C_R colocalization with WGA in the *xz* and *yz* directions relative to the image plane. Scale bar = 10 µm. **(C)** Pearson’s Correlation between the 5-HT_2C_R and WGA for Cys23 and Ser23 was performed on single mid-cell images separate from Z-stack analysis (see Supplementary Figure 3 for representative images with individual channels, colocalization, and scatter plots). Each experiment was performed on five biological replicates with 20–30 fields of view per experiment and 40–70 cells analyzed for each receptor variant. **(D)** Plasma membrane-enriched (n = 7) 5-HT_2C_R protein expression normalized to β-actin in stably expressing Cys23 and Ser23 5-HT_2C_R-CHOp38 cells. Representative virtual blot-like images (left) and electropherograms adjusted to baseline (right) were generated by the Simple Western Compass software using the area under the curve for the peak of interest. Full length electropherograms and virtual blot-like images are located in Supplementary Figure 2. *p < 0.05 vs. Cys23 5-HT_2C_R-CHOp38.

Extracellular N-terminus SNPs within class A GPCRs are reported to demonstrate lower surface expression, alter constitutive activity and half-life of the receptor^45,46^. To test the hypothesis that the Cys23Ser SNP impacts 5-HT_2C_R plasma membrane localization, dual-labeled immunocytochemistry was used to detect colocalization of the 5-HT_2C_R with the plasma membrane marker wheat germ agglutinin (WGA) in cells expressing the Cys23 allele or Ser23 allele. Representative confocal series photomicrographs of the 5-HT_2C_R and WGA staining for the Cys23 allele (Fig. 3A) and the Ser23 allele (Fig. 3B) are provided. In the Cys23 allele-expressing cells, the 5-HT_2C_R (Fig. 3A; green) localized to a greater extent at the plasma membrane (Fig. 3A; red) as emphasized by the orthogonal views of the *xz* and *yz* axis demonstrating the colocalization (Fig. 3A; yellow) of the 5-HT_2C_R and WGA at the plasma membrane. In the Ser23 allele-expressing cells, the 5-HT_2C_R (Fig. 3B; green) demonstrates very little colocalization with WGA (Fig. 3B; red) evidenced by a diminished pattern of the 5-HT_2C_R and WGA colocalization (Fig. 3B; yellow) at the plasma membrane. Quantification of colocalization for the 5-HT_2C_R with WGA in the Cys23 allele- and Ser23 allele-expressing cells was performed on single mid-cell confocal images^47,48^ and are represented as a Pearson’s correlation^49^ (Fig. 3C). The Cys23 allele-expressing cells had significantly higher colocalization with WGA versus the Ser23 allele-expressing cells (Fig. 3C, t(6) = 2.16, p < 0.05). Representative single mid-cell photomicrographs of the 5-HT_2C_R in the Cys23 allele-expressing cells (Supplementary Fig. 3A; green), WGA (Supplementary Fig. 3A; red) and colocalization of the 5-HT_2C_R with WGA (Supplementary Fig. 3A; yellow) are provided. Representative single mid-cell photomicrographs of the 5-HT_2C_R in the Ser23 allele-expressing cells (Supplementary Figure 3B; green), WGA (Supplementary Figure 3B; red) and colocalization of the 5-HT_2C_R with WGA (Supplementary Figure 3B; yellow) are provided. Scatter plots demonstrating the level of 5-HT_2C_R (green pixel intensity), WGA (red pixel intensity), and colocalization (yellow pixel intensity) demonstrate less colocalization between Ser23 allele-expressing cells and WGA relative to Cys23 allele-expressing cells and WGA (Supplementary Figure 3A,B).

The different profile of plasma membrane levels between the Cys23 allele- and Ser23 allele-expressing cells was next assessed using the Wes^TM^ automated Western blotting system^13,50^ of plasma membrane-enriched protein fractions^13,14,51,52^ isolated from the Cys23 5-HT_2C_R-CHOp38 cells or Ser23 5-HT_2C_R-CHOp38 cells. Levels of 5-HT_2C_R plasma membrane-enriched protein expression were lower in cells expressing the Ser23 allele versus the Cys23 allele (Fig. 3D and Supplementary Fig. 2B, t(12) = 2.01, p < 0.05). Levels of 5-HT_2C_R in the cytoplasmic protein fraction were not significantly different between the Cys23 allele- (0.826 ± 0.15 A.U.) and Ser23 allele-expressing cells (0.72 ± 0.03 A.U., n.s.). Surface biotinylation studies followed by Western blot analysis showed surface 5-HT_2C_R protein expression was less in cells stably expressing the Ser23 versus the Cys23 allele (Supplementary Fig. 4). No difference in non-surface 5-HT_2C_R expression or total 5-HT_2C_R expression was detected between Cys23 and Ser23 5-HT_2C_R-CHOp38 cells. Thus, the Ser23 variant directly influences the localization of the 5-HT_2C_R at the plasma membrane versus the wild-type Cys23.

### Inhibition of O-linked glycosylation reduces colocalization of the Ser23 variant with the Golgi apparatus

Single nucleotide polymorphisms in the N-terminus of GPCRs alter the pattern of glycosylation, a post-translational modification that influences receptor function and localization^45,46^. The human Cys23 5-HT_2C_R contains N-linked oligosaccharides and the addition of the N-terminus Ser23 allele could impact the glycosylation pattern of the human 5-HT_2C_R by introducing a new site for N-linked or O-linked glycosylation. *In silico* analysis using NetNGlyc 1.0 (predicts N-glycosylation at Asn-X-Ser/Thr) and NetOGlyc 4.0^53^ (predicts O-linked glycosylation of Ser and Thr residues) software was performed to generate hypotheses regarding the probability of N-linked and O-linked glycosylation of the 5-HT_2C_R expressing the Cys23 or the Ser23. The addition of the Ser23 allele did not alter the probability of N-linked glycosylation at the N-terminus predicted sites but did increase the probability of O-linked glycosylation at the N-terminus. The 5-HT_2C_R expressing the Ser23 allele was predicted to possess a higher probability of O-linked glycosylation at Ser10 (Ser23 = 0.021; Cys23 = 0.007), Ser23 (Ser23 = 0.097; Cys23 = no site), Ser26 (Ser23 = 0.325; Cys23 = 0.191), and Ser28 (Ser23 = 0.426; Cys23 = 0.306) versus the 5-HT_2C_R expressing the Cys23 allele. While none of the predicted Ser23 sites exceeded 0.5 (cut off for high probability of glycosylation), all sites do show an increase in the probability of O-linked glycosylation suggesting that O-linked glycosylation could be altered by the presence of the Ser23 allele.

**Figure 4:**
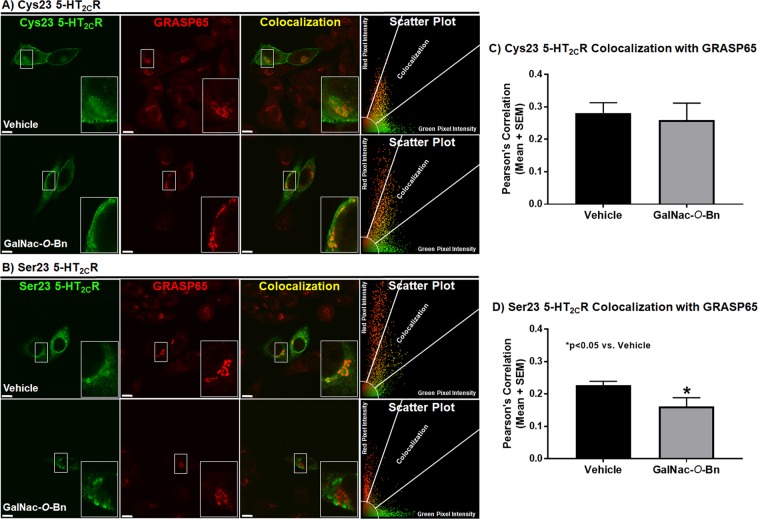
GalNac-*O*-Bn treatment decreases colocalization of the Ser23 allele with GRASP65 with no effect on the Cys23 allele. Colocalization of transiently transfected CHOp38 cells expressing **(A)** Cys23 or **(B)** Ser23 5-HT_2C_R in green with the Golgi reassembly-stacking protein of 65 kDa (GRASP65) in red treated with vehicle or 2 mM Benzyl 2-acetamido-2-deoxy-α-D-galactopyranoside (GalNAc-*O*-Bn) and represented as a single mid-cell slice. The scatter plot represents the type and intensity of pixels for the evaluated cell. Scale bar = 10 µm. Pearson’s Correlation between the 5-HT_2C_R and the GRASP65 for **(C)** Cys23 and **(D)** Ser23 CHOp38 cells. Each experiment was performed on four to eight biological replicates with 20–30 fields of view per experiment and 40–70 cells analyzed for each condition per receptor variant. *p < 0.05 vs. Vehicle.

We next investigated colocalization of the 5-HT_2C_R variants within the Golgi apparatus due to the presence of GalNac transferase and O-glycosylation in the Golgi apparatus^54–59^ and the knowledge that O-glycosylation impacts protein folding and structure (for review^60^) as well as possibly localization and targeting of proteins^61–63^ to support the hypothesis that O-glycosylation could be a part of protein quality control for the 5-HT_2C_R and, thus, impacted by the Cys23Ser SNP. We tested this hypothesis by incubating Cys23 or Ser23 5-HT_2C_R expressing cells with 2 mM Benzyl 2-acetamido-2-deoxy-α-D-galactopyranoside (GalNac-*O*-Bn), a broad range competitive GalNac transferase inhibitor^61,62,64–66^, and then analyzed 5-HT_2C_R colocalization with the cis-Golgi apparatus marker GRASP65 (Golgi reassembly-stacking protein of 65 kDa)^67^. Representative single mid-cell photomicrographs of the 5-HT_2C_R in the Cys23 allele-expressing cells (Fig. 4A; green), GRASP65 (Fig. 4A; red) and colocalization of the 5-HT_2C_R with GRASP65 (Fig. 4A; yellow) are provided. Scatter plots demonstrating the level of 5-HT_2C_R (green pixel intensity), GRASP65 (red pixel intensity), and colocalization (yellow pixel intensity) demonstrated no differences in colocalization between vehicle and GalNac-*O*-Bn treated cells in the Cys23 allele-expressing cells (Fig. 4A). Representative single mid-cell photomicrographs of the 5-HT_2C_R in the Ser23 allele-expressing cells (Fig. 4B; green), GRASP65 (Fig. 4B; red) and colocalization of the 5-HT_2C_R with GRASP65 (Fig. 4B; yellow) are provided. Scatter plots for the GalNac-*O*-Bn treated Ser23 allele-expressing cells demonstrated decreased levels of colocalization between the 5-HT_2C_R and GRASP65 versus vehicle (Fig. 4B). Quantification of colocalization (yellow pixel intensity) for the 5-HT_2C_R with GRASP65 in the Cys23 allele- and Ser23 allele-expressing cells was performed on single mid-cell confocal images^47,48^ and are represented as a Pearson’s correlation^49^. A two-way ANOVA comparing GalNac-*O*-Bn treatment and the different 5-HT_2C_R alleles revealed a significant genotype effect only (F(2, 19) = 5.52, p < 0.05), indicating that genotype had a similar effect on treatment status. Subsequent analysis of the Cys23 allele-expressing cells showed no significant difference between vehicle and GalNac-*O*-Bn treated cells (Fig. 4C, n.s.). GalNac-*O*-Bn treatment decreased colocalization of the Ser23 allele with GRASP65 (Fig. 4D, t(9) = 2.63, p < 0.05) suggesting that the Ser23 allele may have O-linked glycosylation that impacts its subcellular localization.

### Serotonin treatment decreases the Ser23 5-HT_2C_R localization within the recycling pathway versus the Cys23 5-HT_2C_R

**Figure 5:**
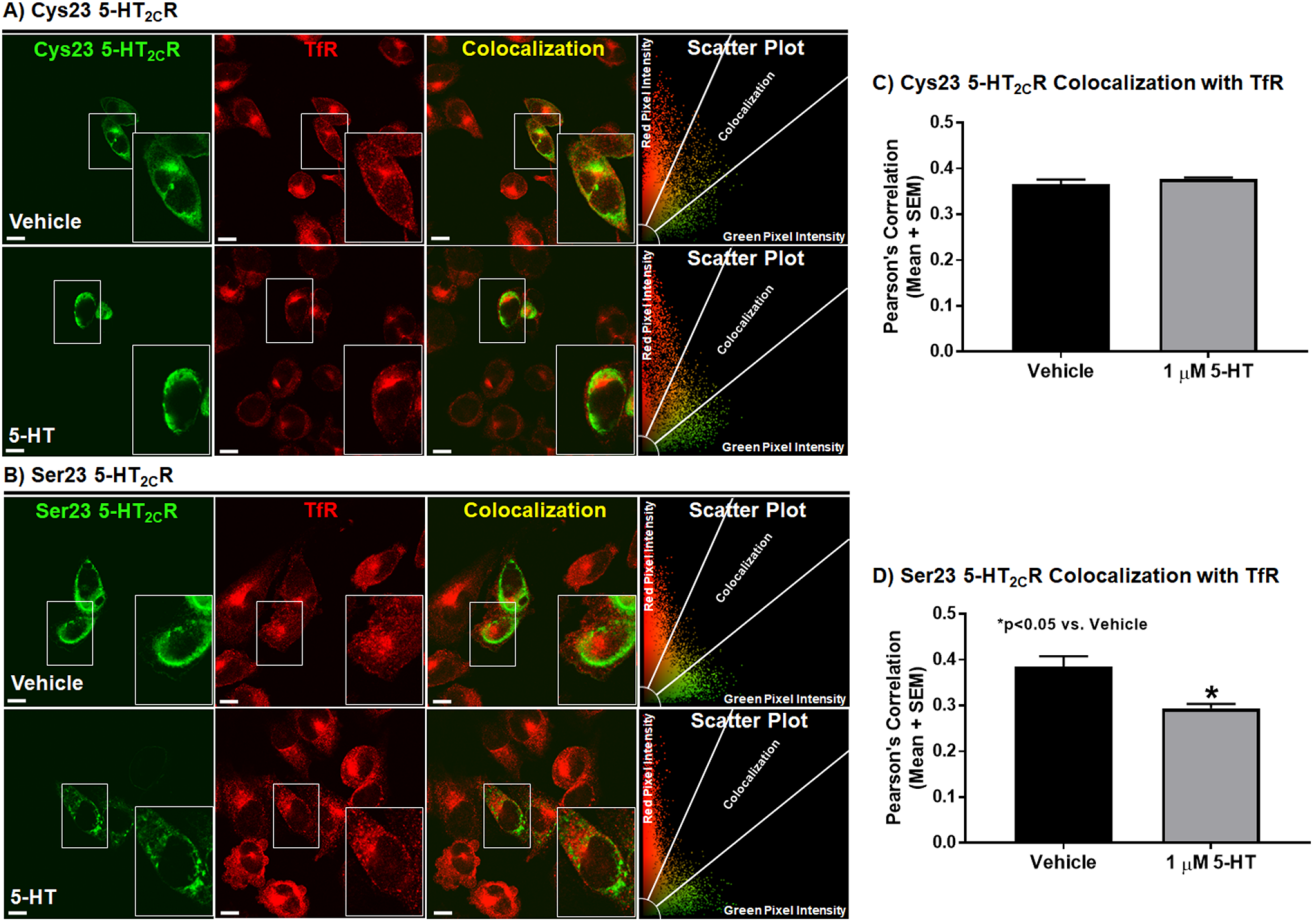
Serotonin treatment decreases colocalization of the Ser23 allele with TfR with no effect on the Cys23 allele. Colocalization of transiently transfected CHOp38 cells expressing **(A)** Cys23 or **(B)** Ser23 5-HT_2C_R in green with the Transferrin Receptor (TfR) in red treated with vehicle or with 1 µM 5-HT for 60 min and represented as a single mid-cell slice. The scatter plot represents the type and intensity of pixels for the evaluated cell. Scale bar = 10 µm. Pearson’s Correlation between the 5-HT_2C_R and the TfR for **(C)** Cys23 and **(D)** Ser23 CHOp38 cells. Each experiment was performed on three biological replicates with 25–30 fields of view per experiment and 40–70 cells analyzed for each condition per receptor variant. *p < 0.05 vs. Vehicle.

The functional response of a GPCR is regulated by its faithful movement through the receptor recycling pathways; this process also controls the availability of the cell surface 5-HT_2C_R to bind ligands and initiate downstream intracellular signaling^35–37^. The actual impact of the Cys23Ser SNP on 5-HT_2C_R localization through the recycling pathway is not known. Our CHOp38 cells stably express synaptophysin/p38 and transferrin receptor (TfR)^42^. The TfR undergoes recycling constitutively, in the presence or absence of transferrin (Tfn) binding, and is a useful way to track the bulk plasma membrane traversing the receptor recycling pathway^68^. Further, synaptophysin/p38 colocalizes with internalized Tfn and the TfR on early endosomes^42,69^. Thus, TfR localization can be used as a marker for the recycling pathway in CHOp38 cells. Immunocytochemical analyses to detect colocalization of the 5-HT_2C_R with the TfR was performed in cells expressing the Cys23 allele or Ser23 allele. Representative single mid-cell photomicrographs of the 5-HT_2C_R in the Cys23 allele-expressing cells (Fig. 5A; green), TfR (Fig. 5A; red) and colocalization of the 5-HT_2C_R with the TfR (Fig. 5A; yellow) treated with vehicle or 1 µM 5-HT (60 min) are provided. Scatter plots of the level of 5-HT_2C_R (green pixel intensity), TfR (red pixel intensity), and colocalization (yellow pixel intensity) demonstrated no differences between levels of colocalization for the 5-HT_2C_R with the TfR for the Cys23 allele-expressing cells between vehicle or 5-HT treated cells (Fig. 5A). Representative single mid-cell photomicrographs of the 5-HT_2C_R in the Ser23 allele-expressing cells (Fig. 5B; green), TfR (Fig. 5B; red) and colocalization of the 5-HT_2C_R with the TfR (Fig. 5B; yellow) treated with vehicle or 1 µM 5-HT (60 min) are provided. The Ser23 allele-expressing cells had decreased colocalization of the 5-HT_2C_R with the TfR after treatment with 5-HT versus vehicle (Fig. 5B).

Quantification of colocalization (yellow pixel intensity) for the 5-HT_2C_R with the TfR in the Cys23 allele- and Ser23 allele-expressing cells was performed on single mid-cell confocal images^47,48^ and represented as a Pearson’s correlation^49^. A two-way ANOVA comparing 5-HT treatment and the different 5-HT_2C_R alleles revealed a significant genotype effect (F(1, 8) = 5.63, p < 0.05), a significant treatment effect (F(1, 8) = 7.96, p < 0.05) and an interaction (F(1, 8) = 14.37, p < 0.05), suggesting that colocalization between the 5-HT_2C_R and the TfR differed amongst genotype for at least one of the treatments. Further testing showed no significant difference for the Cys23 allele between vehicle and 5-HT treatment (Fig. 5C, n.s.). Serotonin treatment significantly decreased colocalization of the Ser23 5-HT_2C_R with the TfR versus vehicle (Fig. 5D, t(4) = 3.616, p < 0.05). These results demonstrate that comparable levels of the Cys23 and the Ser23 alleles can be detected in TfR-positive recycling compartments and that both the Cys23 5-HT_2C_R and Ser23 5-HT_2C_R enter the recycling pathway. However, upon 5-HT stimulation the Ser23 5-HT_2C_R leaves the recycling pathway while the Cys23 5-HT_2C_R maintains its TfR colocalization.

### Serotonin treatment differentially alters the Cys23 versus Ser23 5-HT_2C_R localization within early endosomes

**Figure 6:**
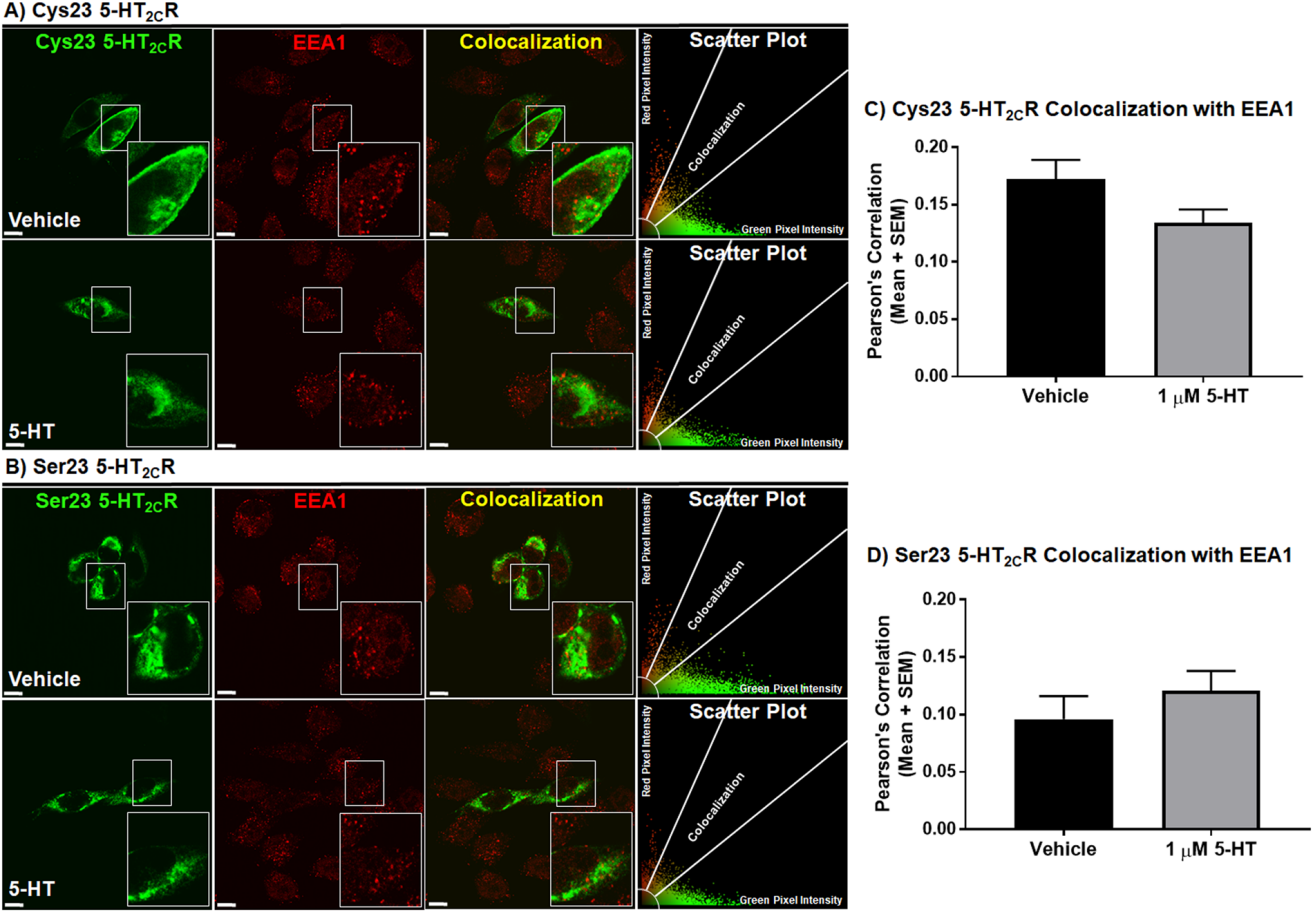
Ser23 allele has decreased colocalization with EEA1 basally versus the Cys23 allele. Colocalization of transiently transfected CHOp38 cells expressing **(A)** Cys23 or **(B)** Ser23 5-HT_2C_R in green with the Early Endosomal Antigen (EEA1) in red treated with vehicle or with 1 µM 5-HT for 60 min and represented as a single mid-cell slice. The scatter plot represents the type and intensity of pixels for the evaluated cell. Scale bar = 10 µm. Pearson’s Correlation between the 5-HT_2C_R and the EEA1 is depicted in bar graphs for **(C)** Cys23 and **(D)** Ser23 CHOp38 cell. Each experiment was performed on three biological replicates with 25–30 fields of view per experiment and 40–70 cells analyzed for each condition per receptor variant.

As the TfR is localized to multiple compartments within the recycling pathway, including the plasma membrane, early and recycling endosomes^69,70^, we wanted to further delineate the profile of the Cys23 5-HT_2C_R and Ser23 5-HT_2C_R localization within a key organelle involved in the initial steps of receptor recycling, i.e., the early endosome. Immunocytochemical analyses to detect colocalization of the 5-HT_2C_R with the early endosome antigen 1 (EEA1) were performed in cells expressing the Cys23 allele or Ser23 allele. Representative single mid-cell photomicrographs of the 5-HT_2C_R in the Cys23 allele-expressing cells (Fig. 6A; green), EEA1 (Fig. 6A; red) and colocalization of the 5-HT_2C_R with EEA1 (Fig. 6A; yellow) treated with vehicle or 1 µM 5-HT (60 min) are provided. Representative single mid-cell photomicrographs of the 5-HT_2C_R in the Ser23 allele-expressing cells (Fig. 6B; green), EEA1 (Fig. 6B; red) and colocalization of the 5-HT_2C_R with EEA1 (Fig. 6B; yellow) treated with vehicle or 1 µM 5-HT (60 min) are provided. Scatter plots of the level of 5-HT_2C_R (red pixel intensity), EEA1 (green pixel intensity), and colocalization (yellow pixel intensity) demonstrated lower levels of colocalization for the 5-HT_2C_R with EEA1 for the Ser23 allele- (Fig. 6B) versus Cys23 allele- (Fig. 6A) expressing cells.

Quantification of colocalization (yellow pixel intensity) for the 5-HT_2C_R with EEA1 in the Cys23 allele- and Ser23 allele-expressing cells was performed on single mid-cell confocal images^47,48^ and represented as a Pearson’s correlation^49^. A two-way ANOVA comparing 5-HT treatment and the different 5-HT_2C_R alleles revealed a significant genotype effect only (F(1, 8) = 7.26, p < 0.05), indicating that genotype had a similar effect on treatment status. Subsequent analysis of Ser23 allele-expressing cells demonstrated significantly less colocalization with EEA1 than the Cys23 allele-expressing cells (Fig. 6, t(4) = 2.916, p < 0.05) basally. Serotonin treatment of the Cys23 allele-expressing cells induced a trend towards a decrease in the colocalization of Cys23 5-HT_2C_R with EEA1 (Fig. 6C, t(4) = 1.869, p = 0.067) with no effect on Ser23 5-HT_2C_R colocalization with EEA1 (Fig. 6D; n.s.). While both the wild-type Cys23 and the Ser23 variant are capable of early endosome internalization, the Ser23 variant is found to a lower degree even after agonist stimulation (Fig. 6D), possibly as a result of less plasma membrane 5-HT_2C_R available for endocytosis.

## Discussion

We discovered that the Ser23 variant demonstrated lower maximum 5-HT-induced ${{{\rm{Ca}}}_{i}}^{++}$ release and a decreased potency versus the wild-type Cys23. Western blot and immunocytochemistry results indicated lower 5-HT_2C_R plasma membrane localization in the Ser23 allele- versus the Cys23 allele-expressing cell lines, with total protein levels equal. Further, O-linked glycosylation of the Ser23 variant, but not the wild-type Cys23, may be a post-translational mechanism which alters its localization within the Golgi apparatus and subsequent localization to the plasma membrane. Subcellular localization studies detected no differences between the Cys23 and Ser23 co-localization with TfR under basal conditions, while less co-localization with EEA1 was observed for the Ser23 versus the Cys23 under basal conditions. Further, the Cys23 allele potentially has lower agonist-induced EEA1 localization but does not exit the recycling pathway as evidenced by no change in colocalization with the TfR. In contrast, the Ser23 variant leaves the recycling pathway (i.e., less agonist-induced TfR colocalization), but there was no agonist-induced effect on EEA1 colocalization. These data suggest that the Cys23 allele has the potential to be recycled and that lower levels of basal plasma membrane expression of the Ser23 variant may indicate less available receptor at the plasma membrane to be internalized after agonist stimulation. Taken together, the Ser23 variant exhibits a distinct pharmacological and subcellular localization profile versus the wild-type Cys23 allele, which could impact aspects of receptor pharmacology in individuals expressing the Cys23Ser SNP.

The ultimate level of functional activity of the 5-HT_2C_R is determined by a culmination of factors, including the number and availability of active pools of receptors at the plasma membrane and effective coupling to and activation of downstream signaling components. Our findings demonstrate that the naturally occurring Ser23 variant imparts profoundly reduced calcium signaling activity (potency and efficacy) as well as lower plasma membrane localization. Studies investigating the impact of the Cys23Ser SNP on 5-HT_2C_R function are few and far between with relatively contradictory results. A decrease in potency, but not efficacy, in the ${{{\rm{Ca}}}_{i}}^{++}$ release assay for cells expressing the Ser23 variant versus the wild-type Cys23 was reported^27^. Intriguingly, signaling through IP_3_ formation is identical between the variants^28^. Transient overexpression of the Ser23 produces higher constitutive activity, agonist-induced desensitization, and differential resensitization time courses and plasma membrane localization patterns^27^, which likely contributes to the observed altered pharmacological responsiveness^29^. Further, the Ser23 variant resensitizes more rapidly following prolonged inverse agonist exposure relative to the wild-type Cys23^29^, possibly due to differences in recovery from constitutive internalization^35,71^. Rapid transmembrane signaling events require cell surface binding of 5-HT to the 5-HT_2C_R to facilitate Gα_q/11_ coupling and the activation of plasma membrane located PLCβ to generate intracellular second messengers IP_3_ and ${{{\rm{Ca}}}_{i}}^{++}$ release^2^. As ligand-mediated G protein-dependent downstream cascades converge to stimulate ${{{\rm{Ca}}}_{i}}^{++}$ release, it is conceivable that the multiple agonist-directed signaling pathways are differentially recruited by the Ser23 variant, as opposed to the wild-type Cys23, suggesting that the discrepancies in the literature may be due to the signaling endpoint analyzed. Further, our discovery of lower plasma membrane targeting for the Ser23 likely also contributes to the deficient calcium signaling observed.

The 5-HT_2C_R contains a 57 amino acid N-terminus tail with two predicted cleavage sites after amino acid 32 or 22^72,73^. Cleavage of the signal peptide is an essential step for the processing of the GPCR from the ER to the Golgi apparatus and ultimately to the plasma membrane; malfunction of this process can result in the intracellular retention of the 5-HT_2C_R during maturation^72,73^. If cleavage of the signal peptide occurs at amino acid 32, the maturation of the 5-HT_2C_R (independent of the allele expressed) as well as subsequent proper targeting to the plasma membrane could be impacted^72,73^. Alternatively, if cleavage at amino acid 22 occurs^72,73^, then the presence of the Ser23 allele and removal of the Cys-dependent disulfide bond in the mature 5-HT_2C_R would have a greater impact on the efficacy of cleavage, maturation, and targeting to the plasma membrane. This is a unique property of the 5-HT_2C_R and warrants future investigation as to the effect on ligand-mediated functional capacity and/or constitutive activity.

The clinical implications of the Cys23Ser SNP suggest an association between expression of the Ser23 variant and phenotypic behaviors. Specifically, subjects who carry the Ser23 exhibit greater dopamine release in striatal regions in response to salient, stressful stimuli^21^; the 5-HT_2C_R regulates dopamine release in the meso-corticoaccumbens circuit, specifically, agonists decrease dopamine release while antagonists increase dopamine release^74–76^. Cocaine-dependent subjects expressing the Ser23 variant have higher cue reactivity as compared to subjects expressing the wild-type Cys23^24^; reduced plasma membrane localization and pharmacological responsiveness of the 5-HT_2C_R corresponds to higher cue reactivity in preclinical studies^11–13^. We discovered that the plasma membrane localization of the Ser23 variant is less and has an altered distribution in the recycling pathway compared to the wild-type Cys23. Our results in stable-expressing cell lines and previous reports using radioligand binding of transiently transfected cells^27,28^ demonstrate similar levels for these two receptor variants (but see^29^), indicating reduced functional activity of the Ser23 is not due to reduced total receptor levels. Taken together, these results may elucidate a potential neurobiological mechanism whereby the Cys23Ser SNP influences the subcellular localization and signaling capacity in the human participants; however, this is unknown at this time. As the functional capacity of the 5-HT_2C_R is most likely brain region dependent and highly responsive in the brain, more detailed *in vitro* studies which investigate differences in pharmacological responsiveness (e.g., constitutive activity, ligand-mediated signaling, desensitization/resensitization processes) are critical not only because of phenotype linkage, but also medication responsiveness in humans.

**Figure 7:**
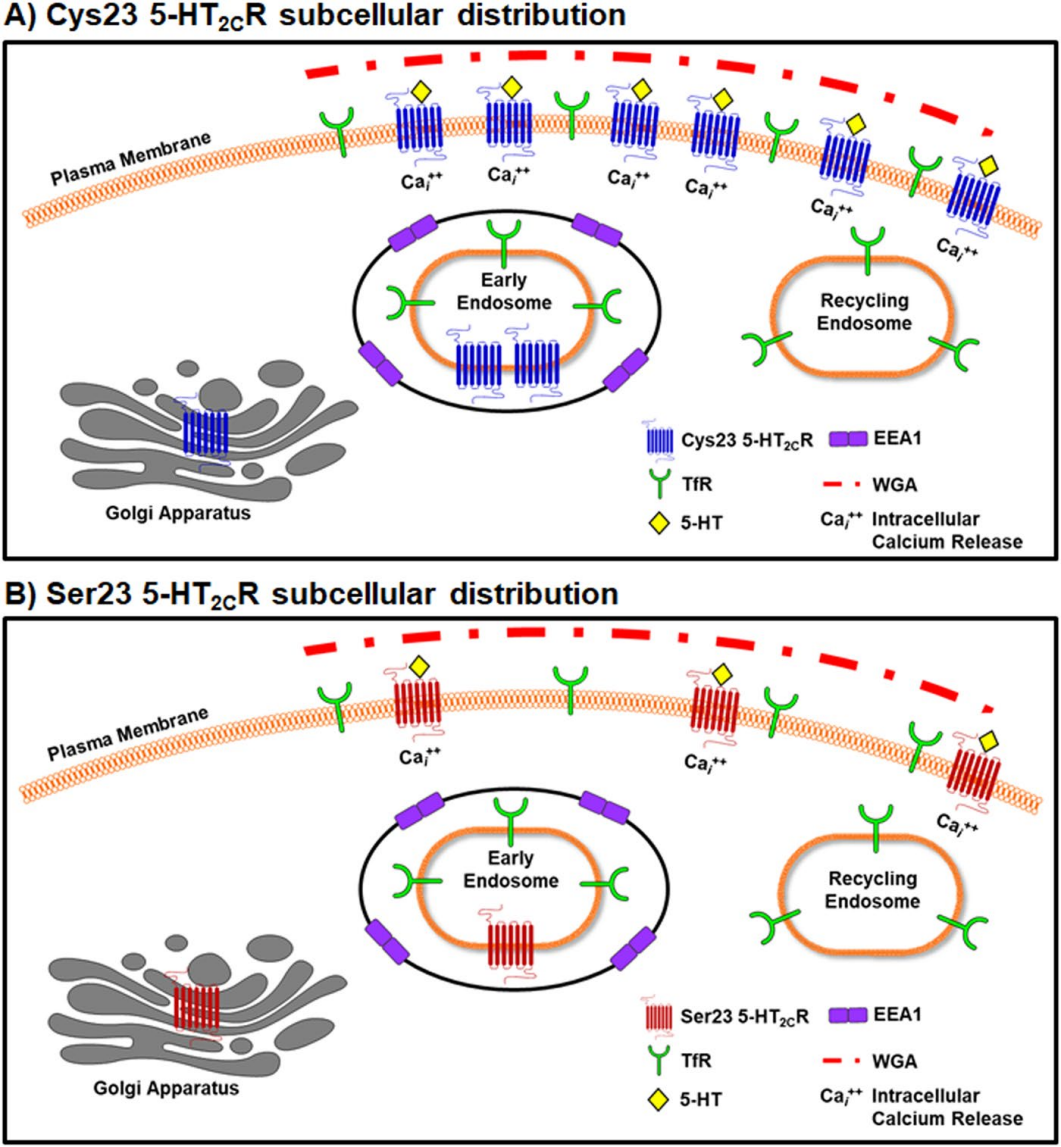
Conceptual framework of the (**A**) Cys23 or (**B**) Ser23 5-HT_2C_R pattern of subcellular distribution and signaling.

In summary, the Cys23Ser SNP alters the subcellular localization and ultimately function of the 5-HT_2C_R (Fig. 7). Changes in the function and localization of the 5-HT_2C_R have strong implications for the use of 5-HT_2C_R ligands in individuals that express the Cys23Ser SNP and we propose future studies in which the function and localization of the Cys23Ser SNP is investigated in the native neuronal environment are warranted. Taken together, a greater appreciation of the full spectrum of genetic mutations and subsequent downstream signal activation is critical when elucidating the functional status of these receptors in neural mechanisms, including the various regulatory features (e.g., subcellular localization) that serve to modulate downstream activation and functionally distinguish 5-HT_2C_R variants actions.

## Methods

### Ligands

Serotonin (5-HT, Acros Organics, Thermo Fisher Scientific, Fair Lawn, NY) was dissolved in 1x HBSS (Cellgro; Invitrogen, Manassas, VA). Benzyl 2-acetamido-2-deoxy-α-D-galactopyranoside (GalNac-*O*-Bn, Sigma-Aldrich, St. Louis, MO) was dissolved in serum free Dulbecco’s modified Eagle’s medium. All purchased compounds for the radioligand binding assay were >98% pure according to the manufacturers. Mianserin hydrochloride (Sigma Aldrich, St. Louis, MO) was dissolved in dimethyl sulfoxide (DMSO) (10 mM) and prepared fresh daily. [^3^H]-Mesulergine (83.0 Ci/mmol) was purchased from PerkinElmer Life Sciences (Waltham, MA).

### Cell culture and transfection

A Chinese Hamster Ovary (CHO) cell line stably transfected with the human Transferrin Receptor (TfR) and the synaptic vesicle protein synaptophysin/p38 (CHOp38)^42^ was maintained at 37 °C and 5% CO_2_ in Dulbecco’s modified Eagle’s medium, containing 7.5% Cosmic calf sera, 5% heat-inactivated horse serum, 100 units/mL penicillin/streptomycin (CHOp38 medium). The Ser23 allele was generated by site directed mutagenesis (QuikChange II Site-Directed Mutagenesis, Agilent Technologies, Santa Clara, CA) from the human Cys23 5-HT_2C_R variant [non-edited isoform (INI); UMR cDNA Resource Center (Rolla, MO)]. Both variants were subcloned into zeocin-resistant pcDNA3.1 and transfected using Fugene® 6 Transfection Reagent (Promega Corporation, Madison, WI) according to the manufacturer’s specifications. CHOp38 cells stably expressing the Cys23 allele or Ser23 allele were selected in CHOp38 medium supplemented with 400 µg/mL zeocin 48 hr post transfection.

### Intracellular calcium release assay

The intracellular calcium (${{{\rm{Ca}}}_{i}}^{++}$) release assay was performed as previously described with minor modifications^40^. Briefly, 150 µL of cells from passages 8–12 were plated in black wall, flat, clear bottom 96-well tissue culture plates at a density of 16,000 cells/well in normal growth media. Care was taken to ensure even plating of cells, including frequent agitation or trituration of cells in the source reservoir; plates were placed on a rotary shaker at low speed for 10 min at room temperature. Approximately 24 hr following plating, the normal growth media was removed, and cells were fed with serum-free (SF) Dulbecco’s modified Eagle’s medium. Following a 3-hr incubation at 37 °C, SF medium was replaced with 40 µL Hank’s balanced saline solution (HBSS; without CaCl_2_ or MgCl_2_, pH 7.4) plus 40 µL Calcium 4 dye solution (FLIPR No-wash kit R8142, Molecular Devices, Sunnyvale, CA) supplemented with 2.5 mM of water-soluble probenicid (Sigma, St. Louis, MO) to inhibit extracellular transport of the dye. Plates were incubated for 60 min at 37 °C followed by 30 min at 25 °C in the dark.

Calcium-induced fluorescence signal (ex = 485 nm, em = 525 nm) was measured with a FlexStation 3 instrument (Molecular Devices). A baseline was established for each well during the initial component of each run. Addition of 20 µL of 5X concentrated 5-HT or vehicle (HBSS) occurred at 17 sec, and fluorescence was recorded every 1.7 sec for 60 sec to determine agonist activity. Maximum peak heights were determined by the SoftMax software (Pro 5.4.5; Molecular Devices) for each well. After the final readings, cells were fixed in 2% paraformaldehyde (Sigma) for 45 min at room temperature. After fixation, cells were rinsed with water, air dried, and 50 µL of filtered crystal violet solution (0.1% in water) was added for 10 min at 25 °C, and the wells were rinsed again. Absorbance in 50 µL of 10% acetic acid was read at 590 nm. Peak ${{{\rm{Ca}}}_{i}}^{++}$ release responses from each well were normalized to total cell mass as determined with crystal violet staining, a value proportional to cell mass that can be used as an estimate of cell number^40^. The responses were then normalized to the average 1 µM 5-HT response in the Cys23 5-HT_2C_R-CHOp38 (Clone 1) to give a percent response. Each experiment was performed in technical triplicates with two to six biological replicates.

### Saturation binding assay

Stably transfected Cys23 5-HT_2C_R-CHOp38 and Ser23 5-HT_2C_R-CHO-p38 cells were scraped from plates and collected by centrifugation at 4000 g at 4 °C for 25 mins in ice cold assay buffer containing 50 mM Tris HCl, 10 mM MgCl_2_ and 0.1 mM EDTA. Samples were collected by centrifugation three times at 4500 g at 4 °C for 20 mins and were used fresh for binding assays. Saturation binding isotherms were performed in 96-well plates using similar methods as previously reported^77^. For saturation binding assays, 0.02 to 15 nM of [^3^H]-mesulergine (PerkinElmer, Waltham, MA) was used to obtain affinity (K_D_) and receptor concentration (B_MAX_) values. Non-specific binding was determined in the presence of 10 µM of mianserin hydrochloride (Sigma Aldrich, St. Louis MO). The reaction mixtures were incubated at 25 °C for 90 mins on a plate shaker in the dark to reach equilibrium, and then passed rapidly through a printed filtermat soaked in 0.5% polyethylenimine using a FilterMate Harvester (PerkinElmer, Waltham, MA). The printed filtermat containing bound [^3^H]-mesulergine was microwaved for one minute to dry, then a MeltiLex sheet was melted onto the printed filtermat via a hotplate. The contents were sealed and counted for scintillation using a MicroBeta 2 (PerkinElmer, Waltham, MA). Direct radioligand concentrations were measured by pipetting into 1 mL of Optiphase Supramix (PerkinElmer, Waltham, MA) and measured on a Tri-Carb 2910TR liquid scintillation analyzer (PerkinElmer, Waltham, MA). Protein concentrations were determined using the bicinchoninic acid (BCA) protein assay kit (Thermo Scientific, Waltham, MA) by measuring absorbance values (562 nm) on a H4 synergy reader (Biotek, Winooski, VT). Each experiment was performed in technical triplicates with three biological replicates.

### Protein extraction and immunoblotting

Plasma membrane-enriched and cytoplasmic protein fractions were prepared via differential centrifugation as previously described with minor modifications^13,50^. This well-established differential centrifugation method *enriches for the plasma membrane* and will also contain membranes from other organelles such as the ER and Golgi apparatus. However, this fraction still contains a majority of plasma membrane and has been used numerous times to show changes in plasma membrane expression of proteins^13,14,51,52^. Cells (~3–7 × 10^7^) were homogenized in 300–600 µL of extraction buffer [(10 mM HEPES, 1 mM EDTA, 2 mM EGTA, 1 mM dithiothreitol (DTT), 10 mM MgCl_2_), plus protease inhibitor cocktail and phosphatase inhibitor 2 and 3 cocktails (10 µL/mL; Sigma–Aldrich, St. Louis, MO)]. The homogenate was centrifuged at 1,000 g for 10 min at 4 °C to pellet the nuclear fraction. The supernatant (S1) was collected and an aliquot was saved as the total homogenate (TH) fraction, the remaining TH fraction was centrifuged at 15,000 g for 30 min at 4 °C to pellet the plasma membrane-bound enriched protein fraction (P2). The supernatant (S2) was collected and centrifuged at 20,000 g for 80 min at 4 °C to pellet the plasma membrane-bound enriched protein fraction (P3). The plasma membrane-enriched pellets were resuspended in buffer [20 mM HEPES, 200 mM NaCl, 1 mM EDTA, 1 mM EGTA, 1 mM DTT, 10 mM MgCl_2_, protease inhibitor cocktail and phosphatase inhibitor 2 and 3 cocktails (10 µL/mL)] plus 1% NP40. The two plasma membrane-bound enriched protein fractions were combined to give the crude plasma membrane fraction. The cytoplasmic fraction was collected and reserved. All protein fractions were stored at −20 °C until use.

For the subcellular localization studies, plasma membrane-enriched, cytoplasmic and total homogenate fractions were assessed using the Wes^TM^ automated Western blotting system (ProteinSimple, San Jose, CA) according to the manufacturers specifications. Wes^TM^ reagents (biotinylated molecular weight marker, streptavidin–HRP fluorescent standards, luminol-S, hydrogen peroxide, sample buffer, DTT, stacking matrix, separation matrix, running buffer, wash buffer, and matrix removal buffer, secondary antibodies, antibody diluent, and capillaries) were obtained from the manufacturer and used with minor modifications^13,50^. The mouse monoclonal 5-HT_2C_R^78^ (D-12; sc-17797 Santa Cruz; 1:10), mouse monoclonal transferrin receptor (TfR, CD71; 136890 Invitrogen; 1:10,000), mouse monoclonal synaptophysin (p38, MAB329, 1:7500, Millipore) and rabbit monoclonal β-actin antibodies (8457, Cell Signaling Technology, 1:50) were diluted with ProteinSimple antibody diluent. Equal amounts of protein (2 µg for MAB329 or 4 µg for all other antibodies) were combined with 0.1X sample buffer and 5X master mix (200 mM DTT, 5X sample buffer, 5X fluorescent standards), gently mixed, and then denatured at 90 °C for 10 min. The denatured samples, biotinylated ladder, antibody diluent, primary antibodies, horseradish peroxidase (HRP) conjugated secondary antibodies, chemiluminescent substrate, and wash buffer were dispensed to designated wells in a pre-filled microplate. Separation electrophoresis (375 V, 28 min, 25 °C) and immunodetection in the capillaries were fully automated using the following settings: separation matrix load for 200 sec, stacking matrix load for 20 sec, sample load for 12 sec, antibody diluent for 30 min, primary antibody incubation for 60 min, secondary antibody incubation for 30 min, and chemiluminescent signal exposure for 5, 15, 30, 60, 120, 240, and 480 sec. Data analyses were performed using the Compass Software (ProteinSimple). Representative “virtual blot” electrophoretic images were automatically generated by the Compass Software (ProteinSimple). Each experiment was performed in technical triplicates with three to seven biological replicates.

### Surface biotinylation and Immunoblotting

Cell surface biotinylation protocol was modified from the Baratta *et al*.^79^ protocol for isolating plasma membrane 5-HT_2C_R protein expression using the Pierce Cell Surface Protein Isolation Kit (Thermo Scientific, Waltham, MA, Cat # 89881). Five flasks of Cys23 or Ser23 5-HT_2C_R stably transfected CHOp38 cells were grown to 70% confluency and were washed twice with cold PBS on ice. Contents of 1 vial Sulfo-NHS-SS-Biotin was dissolved in 48 mL ice cold PBS (ph7.4) to create the biotin solution. Biotin solution (2 mg/mL) was added to flasks and incubated for 1 hr at 4 °C on an orbital shaker. The reaction was quenched using the quenching solution at 500 µL/flask for 20 min on ice. The cells were scraped into the solution from each flask and placed into a 50 mL centrifuge tube. Flasks were then rinsed with a total of 10 mL of Tris Buffered Saline (TBS) and added to the 50 mL centrifuge tube. Cells were centrifuged at 500 g for 3 min at 4 °C and the supernatant was removed. These cells were then resuspended in 5 mL of ice cold TBS and centrifuged at 500 g for 3 min at 4 °C. The supernatant was removed and this step was repeated one additional time. The cellular pellet was stored at −80 °C.

On the day of the assay, each pellet was thawed on ice and 250 µL of homogenization buffer [50 mM Tris-Cl,150 mM NaCl, pH 8.0, protease inhibitor cocktail and phosphatase inhibitor 2 and 3 cocktails (10 µL/mL)] with 0.25% NP40 was added to each pellet. The lysed cells were then centrifuged at 1,000 g for 20 min at 4 °C and the supernatant #1 was collected. The Homogenization buffer with 0.25% NP40 (250 µL) was then added to the cellular pellet and mixed vigorously via vortexing, motorized pestle and trituration then allowed to incubate for 30 min at 4 °C vortexing every 5 min. An aliquot of the solution (50 µL) was saved as a whole cell fraction (i.e. total fraction) and stored at −20 °C. Thus, the total fraction (Supplementary Fig. 4) is taken from whole cell lysate pellet just after lysis. The remaining 200 µL was centrifuged at 1000 g for 2 min at 4 °C and the supernatant was collected and combined with supernatant #1 and stored at −80 °C. Protein concentrations were determined using the BCA protein assay kit.

To set up isolation of labeled proteins, 150 uL NutrAvidin agarose was added to new tubes and briefly centrifuged (1,000 g at 25 °C). The supernatant was discarded and the beads were washed 3x with Homogenization buffer by discarding the supernatant following a 1,000 g spin for 2 min at 25 °C. The supernatant was then thawed and 1 mg of protein was loaded onto the NutrAvidin agarose, sealed with parafilm and allowed to incubate on a carousel for 18 hr at 4 °C. The protein bound NutrAvidin agarose was then centrifuged at 1,000 g for 2 min at 4 °C and the supernatant was transferred to a new tube (non-surface fraction) and stored at −80 °C. The non-surface fraction is the flow through (Supplementary Fig. 4), it is all proteins not surface biotinylated, and thus, did not bind to the NutrAvidin agarose. The NutrAvidin agarose was next washed 3x with 200 µL Homogenization buffer at 1,000 g at 25 °C, discarding the supernatant each time. Protein was eluted from the NutrAvidin agarose by vortexing with 80 µL of SDS-PAGE sample buffer (Thermo Scientific, Waltham, MA, Cat # 39001) with 50 mM DTT added to each column. Each tube was sealed with parafilm and incubated at 25 °C on a carousel for 1 hr. The tubes were then centrifuged for 2 min at 1,000 g at 25 °C and the supernatant collected (surface fraction). The surface fraction (Supplementary Fig. 4) contains all proteins that were surface biotinylated and eluted from the NutrAvidin agarose.

Analysis of 5-HT_2C_R protein expression in the different fractions was preformed using Western blot analysis. In brief, 35 µg of non-surface fraction, 35 µg total fraction and 45 µL of the surface fraction were denatured at 95 °C for 10 min and loaded onto precast gels (NuPage™ 4–12% Bis-Tris; Invitrogen, catalog # NP0335BOX), transferred to PVDF membranes, (Immun-Blot ® PVDF membranes; Bio-Rad, catalog # 162–0239) and immunoblotted with the goat polyclonal antibody towards the 5-HT_2C_R^78^ (N-19, sc-15081, Santa Cruz; 1: 250) overnight at 4 °C. Membranes were incubated with goat IgG IRDye 680 LT (926–68024, LI-COR, 1:10,000, 2 hr at 25 °C) for detection by Odyssey Imaging System (LI-COR, Lincoln, NE). The experiment was replicated in two biological replicates.

### Immunocytochemistry

Immunocytochemistry was conducted using CHOp38 cells transiently transfected with the Cys23 allele or Ser23 allele grown in chamber slides, as described above. In short, 2 µg of the Cys23 allele or Ser23 allele zeocin-resistant pcDNA3.1 plasmids were transfected into CHOp38 cells using Fugene® 6 Transfection Reagent (Promega Corporation) according to the manufacturer’s specifications; the immunocytochemistry assay started 24 hr post transfection. Where needed GalNac-*O*-Bn was added six hrs post transfection at a 2 mM concentration. Cells were incubated with serum-replete media for one-two hrs with or without 2 mM GalNac-*O*-Bn. Cells used for the TfR and EEA1 colocalization studies were then treated for 60 min with serum free media or 1 µM 5-HT. All cells were then rapidly washed twice in ice-cold PBS and fixed in cold 4% paraformaldehyde (15 min). After fixation, cells were washed twice with PBS, permeabilized with 100 µM digitonin (10 min), and then washed twice with 0.1% PBS-Tween 20 (PBST). The plasma membrane marker wheat germ agglutinin (WGA, Alexa 594 conjugated, W11262, Invitrogen) was added at a concentration of 5 µg/mL in PBS for 10 min at 25 °C to the wells and then washed twice with PBS before digitonin permeabilization. Chamber wells were incubated with 4% normal donkey serum for 60 min to block nonspecific antibody binding, followed by primary antibodies to 5-HT_2C_R (D12–488 directly conjugated to Alexa 488, 1:1000, Santa Cruz), early endosomal antigen (EEA1, AB2900, 1:3000, Abcam), GRASP65 (PA3–910, 1:1000, ThermoFisher) and TfR (ab84036,1:500, Abcam) in 4% normal donkey serum for two hrs at 25 °C and then overnight at 4 °C. The chambers were then removed leaving the gasket still attached to the slide and washed five times in 0.1% PBST. The slide was then incubated with anti-rabbit 594 in 4% normal donkey serum for 60 min at 25 °C. The slide was washed five times in 0.1% PBST, once in PBS and mounted using DAPI-containing mounting medium (Vectasheild, Vector Laboratories, H-1000).

### Confocal microscopy

For each condition 20–30 fields of view containing on average one-four Cys23 allele- or Ser23 allele-expressing cells per field of view were acquired mid-cell and represented as a projection (Fig. 3A,B) or as single cuts (Figs 4–6; Supplementary Fig. 3)^47,48^ with lasers 405, 488 and 561 nm using the Leica True Confocal Scanner SPE and Leica Application Suite Advanced software (Leica Microsystems, Wetzlar, Germany). Photomicrographs were taken with 63X oil immersion lens at a zoom of 1.5, 1024 × 1024-pixel size, a pinhole of 137.13 µm and a scanning speed of 400 Hz. Each experiment was performed on a minimum of three biological replicates with 40–70 cells analyzed for each condition per receptor variant.

### Statistical analyses

The E_max_ was defined in the ${{{\rm{Ca}}}_{i}}^{++}$ assay as the maximum possible ${{{\rm{Ca}}}_{i}}^{++}$ response and potency was determined using the pEC_50_. The E_max_ and pEC_50_ values were calculated using 4-parameter nonlinear regression analysis (GraphPad Prism Version 7.02). Data from saturation binding isotherms are presented as B_MAX_ and K_D_ values, as computed by GraphPad Prism using specific binding with Hill slope nonlinear regression curve-fitting algorithm. Emax, pEC50, B_MAX_, or K_d_ values between the Cys23-CHOp38 and Ser23-CHO-p38 cell lines were analyzed with a Student’s t-test (α = 0.05). The Western blot analysis signal is defined as the area under the curve for the 5-HT_2C_R, synaptophysin/p38 and TfR electropherogram peaks and was normalized to the area under the curve of the β-actin electropherogram peak (ProteinSimple); analyses of protein expression levels between the Cys23 allele and the Ser23 allele were made with a Student’s t-test (α = 0.05) Quantification of immunocytochemistry colocalization was preformed using the Pearson’s correlation statistic on unsaturated images (Supplementary Fig. 3 and 5). The Pearson’s correlation statistic was calculated by the LAS X Life Science software by drawing a region of interest around each expressing cell and the level of yellow pixel intensity (i.e., colocalization) measured. Analyses between the average Pearson’s correlation for 5-HT_2C_R colocalization with WGA in the Cys23 allele- or Ser23 allele-expressing cells were made with a Students t-test (α = 0.05). Analyses between the average Pearson’s correlation for 5-HT_2C_R colocalization with GRASP65, TfR or EEA1 in the Cys23 allele- or Ser23 allele-expressing cells were made with a two-way ANOVA with subsequent *a priori* comparisons using Students t-test as appropriate^80,81^ (α = 0.05).

## Supplementary information

Supplementary Figures

## Data Availability

The datasets generated during and/or analyzed during the current study are available from the corresponding author on reasonable request.
