# Supplementary material for: Serotonin 5-HT2C Receptor Cys23Ser Single Nucleotide Polymorphism Associates with Receptor Function and Localization In Vitro
Source: Sci Rep. 2019 Nov 13;9:16737. doi: 10.1038/s41598-019-53124-2 (PMC6853916; doi:10.1038/s41598-019-53124-2)
Supplement: Supplementary file 1 — Supplementary Figures [file 41598_2019_53124_MOESM1_ESM.pdf]

Serotonin 5-HT_{2C} Receptor Cys23Ser Single Nucleotide Polymorphism Associates with Receptor Function and Localization *In Vitro*

Michelle A. Land[1], Holly L. Chapman[1,2], Brionna D. Davis-Reyes[1], Daniel E. Felsing[1,2], John A. Allen[1,2], F. Gerard Moeller[1,4], Lisa A. Elferink[1,3], Kathryn A. Cunningham[1,2], and Noelle C. Anastasio[1,2]

[1]Center for Addiction Research, [2]Department of Pharmacology and Toxicology, [3]Department of Neuroscience, Cell Biology and Anatomy, University of Texas Medical Branch, Galveston, TX
[4]Department of Psychiatry, Virginia Commonwealth University, Richmond, VA

Corresponding Author:
Noelle C. Anastasio, Ph.D.
UTMB Center for Addiction Research
University of Texas Medical Branch
301 University Blvd.
Galveston, Texas 77555-0615
ncanasta@utmb.edu
409-772-9656

KEY WORDS: Serotonin, 5-HT_{2C} receptor, Single Nucleotide Polymorphism, Cys23Ser, nonsynonymous SNP, Localization, rs6318

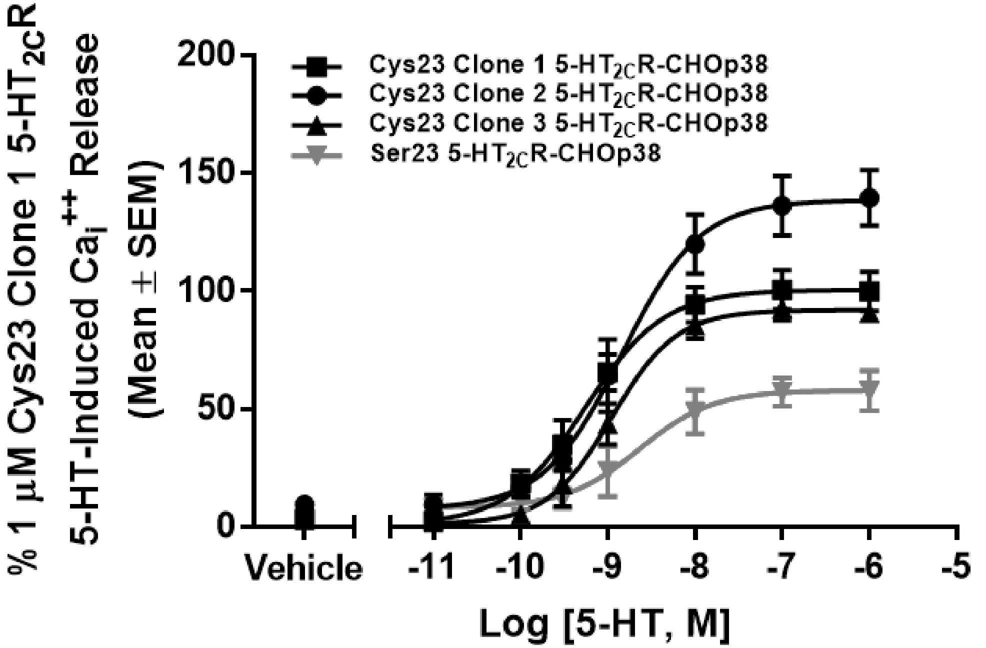

CHOp38 Cell Line	pEC_{50} ± SD	E_{max} ± SD
Cys23 Clone 1	9.24 ± 0.41	101.4 ± 19.16
Cys23 Clone 2	8.81 ± 0.28	137.8 ± 19.95
Cys23 Clone 3	8.96 ± 0.19	91.94 ± 4.56
Ser23	8.54 ± 0.50	57.62 ± 14.83

Supplementary Figure 1. Serotonin-induced intracellular calcium release in stably transfected Cys23-CHOp38 and Ser23-CHOp38 clones. Average traces of a 5-HT-induced concentration-dependent Ca_i^{++} release in Cys23 and Ser23 5-HT_{2C}R-CHOp38 stably-expressing cells (normalized to the average 1 µM 5-HT response in the Cys23 Clone 1 5-HT_{2C}R-CHOp38 to give a percent response). The pEC_{50} and E_{max} (mean ± SD) are from 2 to 6 individual experiments.

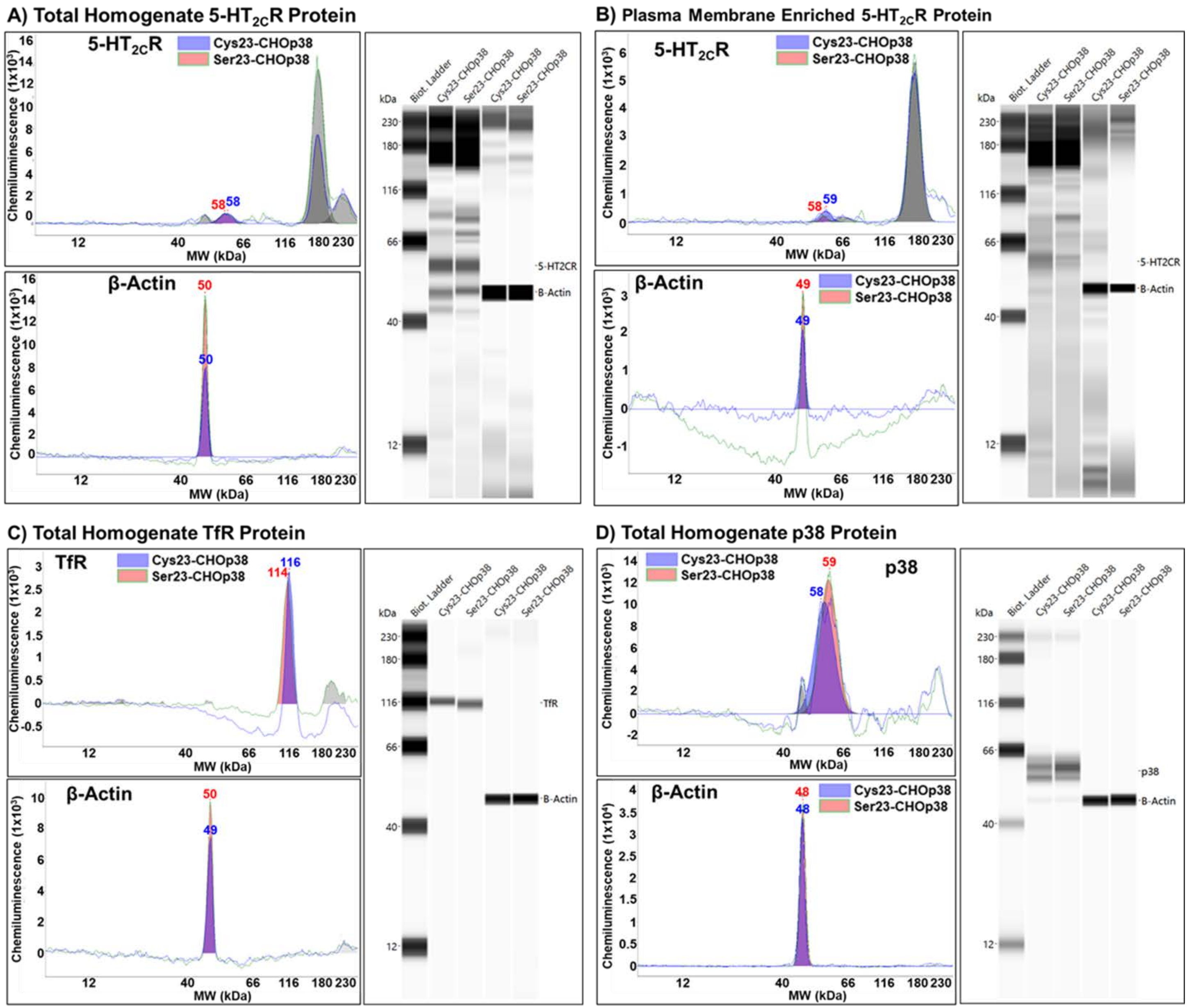

Supplementary Figure 2. Automated capillary-based immunodetection using Wes™ automated Western blotting system from ProteinSimple. Representative electropherograms adjusted to baseline (left) and virtual blot-like images (right) were generated by the Simple Western Compass software using the area under the curve for the peak of interest. Total homogenate **(A)** 5-HT_{2C}R and **(B)** plasma membrane-enriched 5-HT_{2C}R protein in Cys23 and Ser23 5-HT_{2C}R-CHOp38 stably expressing cells. Total Homogenate **(C)** TfR and **(D)** p38 protein expression in Cys23 and Ser23 5-HT_{2C}R-CHOp38 cells.

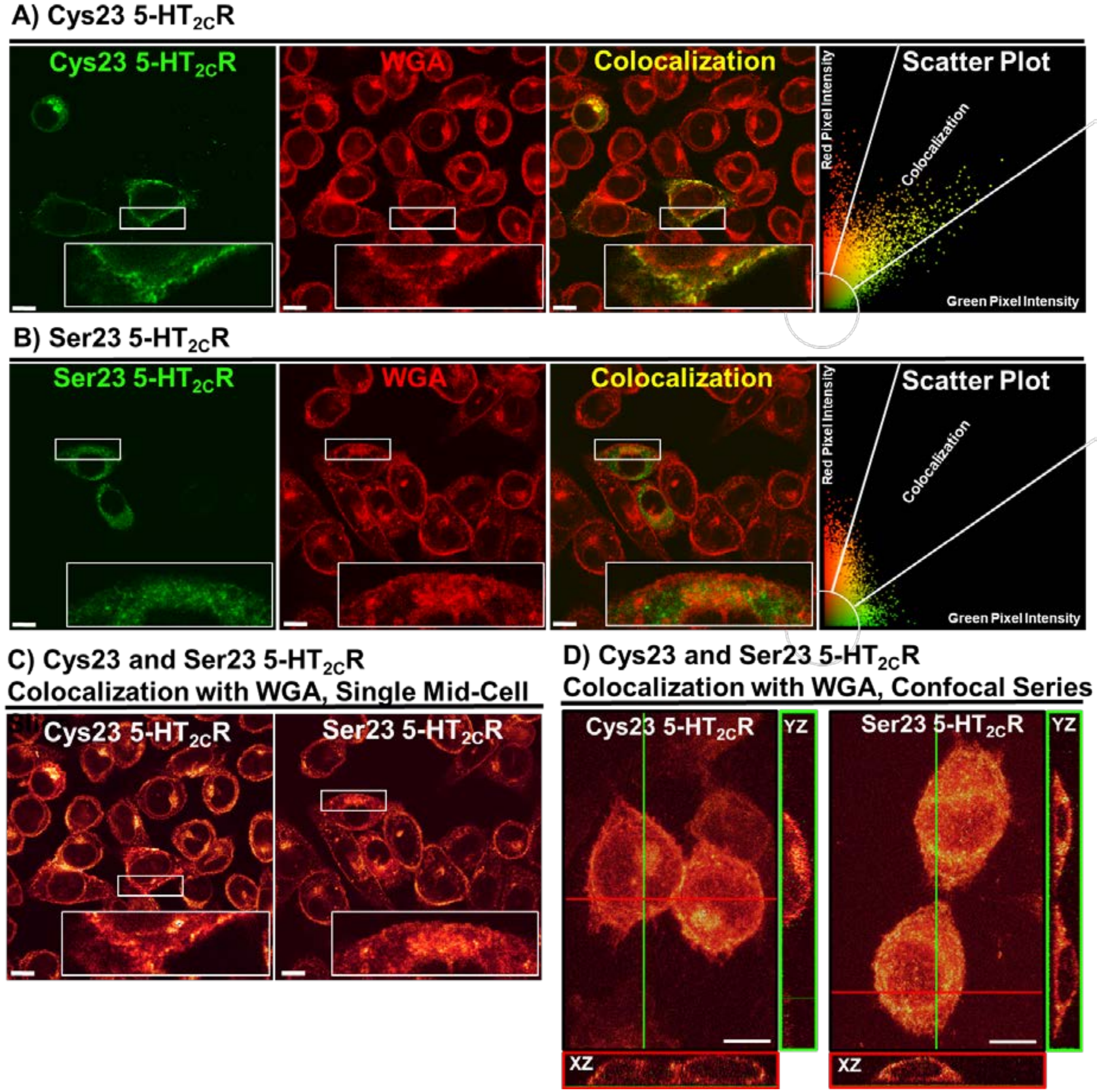

Supplementary Figure 3. Representative images of single or combined channel intensities used for quantifying colocalization or representing colocalization via confocal series. Colocalization of transiently transfected CHOp38 cells expressing **(A)** Cys23 or **(B)** Ser23 5-HT$_{2C}$R in green with the plasma membrane marker wheat germ agglutinin (WGA) in red and represented as a single mid-cell confocal image. The scatter plot represents the type and intensity of pixels for the evaluated cell. Scale bar=10 µm. **(C-D)** Representation of the intensities of the combined red and green channels where intensities range from red to blue, where blue would indicate oversaturation. **(C)** Colocalization of Cys23 or Ser23 5-HT$_{2C}$R with WGA from a single mid-cell confocal image. **(D)** Colocalization images were prepared from a confocal series of Cys23 or Ser23 5-HT$_{2C}$R transfected CHOp38 cells sectioned tangentially. Orthogonal views demonstrate Cys23 or Ser23 5-HT$_{2C}$R colocalization intensities with WGA in the xz and yz directions relative to the image plane. Scale bar=10 µm.

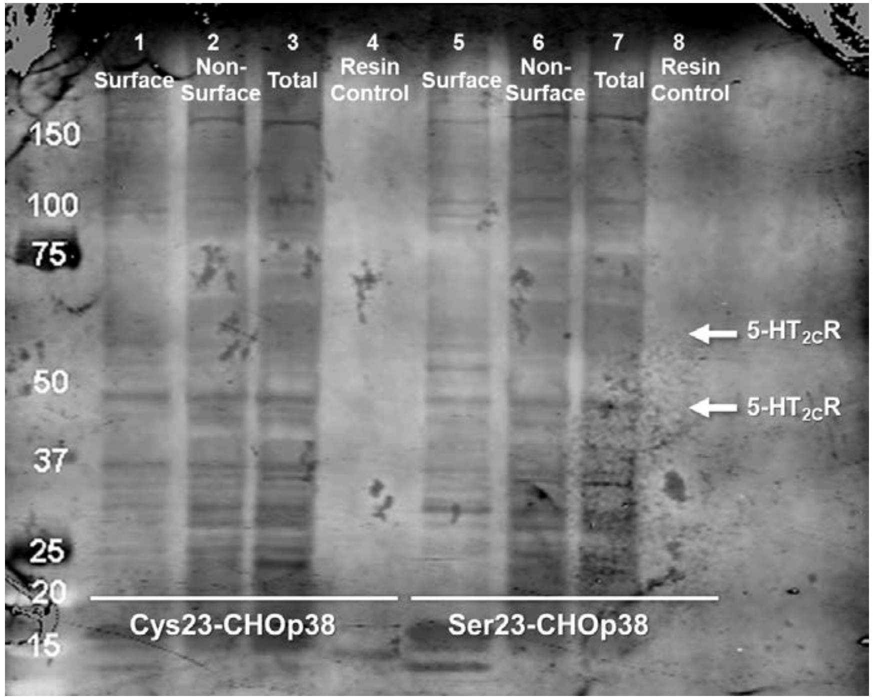

Supplementary Figure 4. Biotinylation of surface proteins from Cys23 and Ser23 5-HT$_{2C}$R-CHOp38 cells. Surface expression of the 5-HT$_{2C}$R protein following biotinylation of surface proteins (protocol adapted from Barratta et al. 2016) and immunoblotting with an N-terminus 5-HT$_{2C}$R antibody (immunoreactive bands denoted by arrows; see Anastasio et al., 2010 for antibody validation). Surface 5-HT$_{2C}$R expression was lower in Ser23-CHOp38 (lane 5) vs. Cys23-CHOp38 (lane 1) stably expressing cells. No differences in non-surface 5-HT$_{2C}$R expression (lanes 2 and 6), total 5-HT$_{2C}$R expression (lanes 3 and 7) or the resin control (lanes 4 and 8) were detected between Cys23 and Ser23 5-HT$_{2C}$R-CHOp38 cells. Results reproduced in an independent biological replicate.

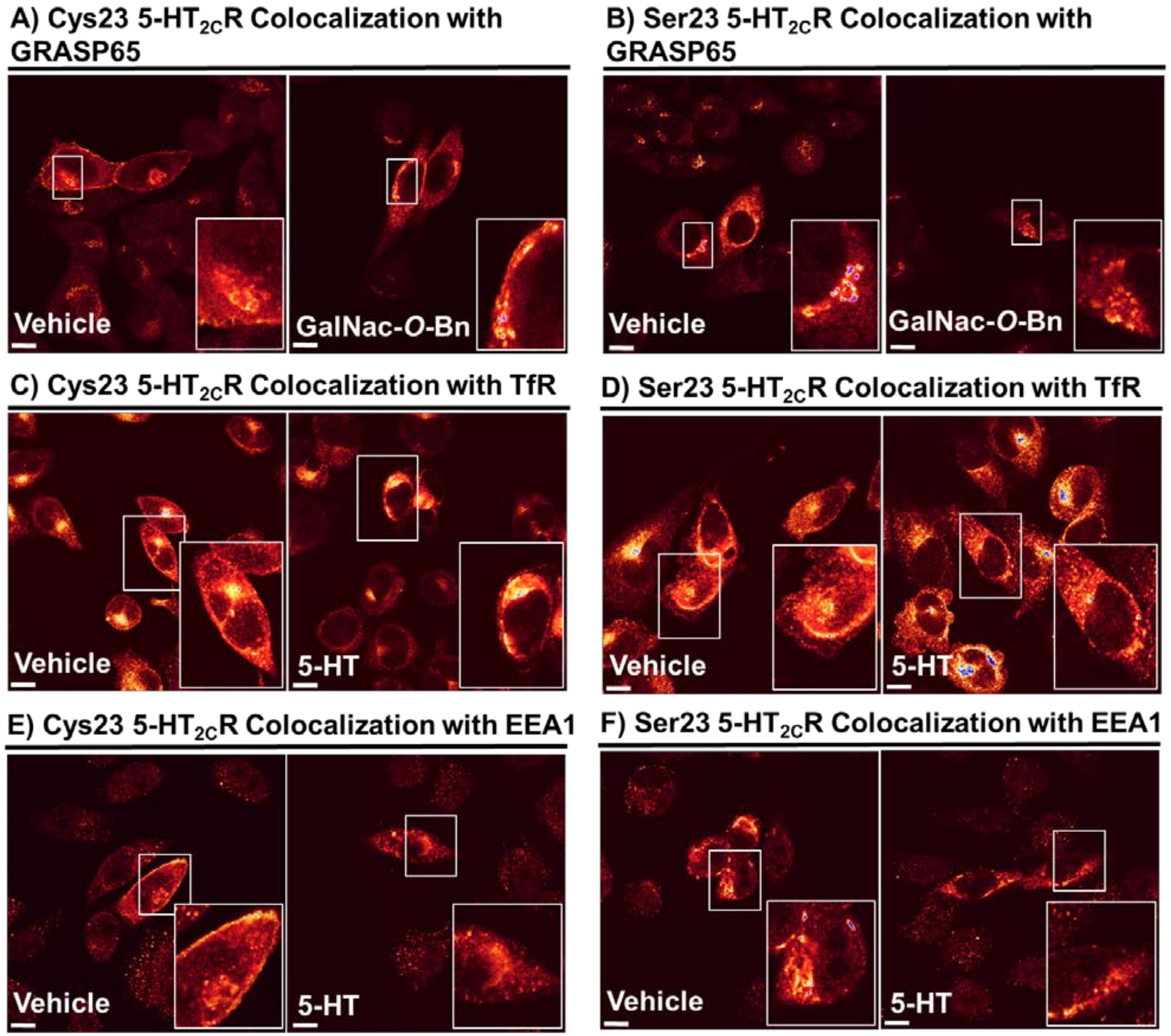

Supplementary Figure 5. Representative images of combined channel intensities used for quantifying colocalization. Colocalization images were prepared from a single tangential confocal image of Cys23 or Ser23 5-HT$_{2C}$R transiently transfected CHOp38 cells. The following images are combined red and green channels only. The intensity increases from red to blue, where blue would indicate oversaturation. Colocalization of **(A)** Cys23 or **(B)** Ser23 5-HT$_{2C}$R with the Golgi reassembly-stacking protein of 65 kDa (GRASP65) treated with vehicle or 2 mM Benzyl 2-acetamido-2-deoxy-α-D-galactopyranoside (GalNAc-*O*-Bn). Colocalization of **(C)** Cys23 or **(D)** Ser23 5-HT$_{2C}$R with the Transferrin Receptor (TfR) treated with vehicle or 1 µM 5-HT for 1 hr. Colocalization of **(E)** Cys23 or **(F)** Ser23 5-HT$_{2C}$R with the Early Endosomal Antigen 1 (EEA1) treated with vehicle or 1 µM 5-HT for 1 hr. Scale bar=10 µm.
